# The impact of telomere-to-telomere genome assembly in the plant pan-genomics era

**DOI:** 10.1270/jsbbs.24065

**Published:** 2025-02-21

**Authors:** Yuta Aoyagi Blue, Hideaki Iimura, Mitsuhiko P. Sato, Kenta Shirasawa

**Affiliations:** 1 Department of Frontier Research and Development, Kazusa DNA Research Institute, 2-6-7 Kazusa-Kamatari, Kisarazu, Chiba 292-0818, Japan

**Keywords:** genome project, long-read sequencing technology, next-generation sequencing technology, telomere-to-telomere sequence

## Abstract

Advances in sequencing technologies have enabled the determination of genome sequences of multiple lines within a single species. Comparative analysis of multiple genome sequences reveals all genes present within a species, providing insight into the genetic mechanisms that lead to the establishment of species. Highly accurate pan-genome analysis requires telomere-to-telomere gapless genome assembly, providing an ultimate genome sequence that covers all chromosomal regions without any undetermined nucleotide sequences. This review describes the genome sequencing technologies and sophisticated bioinformatics required for telomere-to-telomere gapless genome assembly, as well as a genetic mapping that can evaluate the accuracy of telomere-to-telomere genome assembly. Pan-genome analyses may contribute to the understanding of genetic mechanisms not only within a single species but also across species.

## Introduction

Pan-genome analysis is an approach to understanding genetic components in a species by sequencing the genomes of multiple individuals (or cultivars) that cover the genetic diversity of that species (Bayer *et al.* 2020, Schreiber *et al.* 2024). Significant advances in genome sequencing technology over the past 20 years have enabled *de novo* genome assembly of multiple lines (Kong *et al.* 2023, Nurk *et al.* 2022). The completeness of genome assembly can be categorized on three levels: 1) contig-level sequences generated by assembling raw reads from a sequencer; 2) scaffold-level sequences, in which contigs are connected using information about reads that bridge two contigs; and 3) chromosome-level sequences, in which scaffold sequences are aligned on chromosomes of targets (Rhie *et al.* 2021).

In pan-genome analysis, the chromosome-level sequences of multiple lines have been required for analyses of comparative genome structures. This has enabled the detection of variations in chromosome structure (Bayer *et al.* 2020, Schreiber *et al.* 2024), such as translocations and inversions, as well as the presence or absence of gene variations and variations in gene copy number. This approach has resulted in the identification of new genetic components that could not be detected by analysis of a single genome. Pan-genome analysis has enabled the classification of genes into two groups: core and dispensable genes, the latter of which are also known as accessory genes. Core genes are those detected in all individuals analyzed, suggesting that they may be essential for the species, whereas dispensable genes are present in some (not all) individuals of a species.

As of 2021, chromosome-level genome sequences had been reported in more than 100 plant species (Shirasawa *et al.* 2021). However, completeness of the genome sequences varied among plant species because of gaps in which nucleotide sequences were undetermined and/or because of the existence of sequences that could not be assigned to any chromosome. This situation would therefore prevent the definitive determination of the presence or absence of variations, as it is difficult to prove the non-existence of genes.

Most plant genomes are complex, being large in size, with high repeat content and heterozygosity, as well as variations in ploidy. Differences in haplotype are frequently ignored during chromosome-level genome assembly that involves the generation of consensus sequences from two haploid sequences without separating haplotype alleles. Haplotype-phased assembly plays a crucial role in detecting sequence and structure variants and in analyzing allele-specific functions (Zhou *et al.* 2020).

Recent advances in high-quality long-read sequencing methods, together with mate-pair sequencing, linked-read sequencing, Hi-C, optical mapping, and ultra-long reads, are being used more frequently in analyzing plant genomes (Xie *et al.* 2024). The integration of these technologies has enabled the construction of telomere-to-telomere (T2T) haplotype-phased assembly (Nurk *et al.* 2022). High-fidelity (HiFi) long reads (PacBio, Menlo Park, CA, USA), due to their high accuracy and long read lengths, are mostly used for determining the nucleotide sequences of entire genomes, from a telomere on one end to a telomere on the other end of a chromosome without any gaps. Although these HiFi reads can resolve repetitive sequences, repetitive regions longer than HiFi reads remain unresolved. ONT ultra-long reads (Oxford Nanopore Technologies, Oxford, UK) can help resolve the repetitive sequences in plant genomes with high repetitive contents, although at relatively lower accuracy. This review describes recent advances in plant pan-genome analysis, including the introduction of sequencing and bioinformatic technologies that enable T2T-level genome assembly in plant species.

## Classical genome sequencing in plants

Genome sequencing in higher plants started with Arabidopsis (The Arabidopsis Genome Initiative 2000), a member of the Brassicaceae. Arabidopsis has been recognized as a model for plant research because of its small-sized genome, rapid life cycle, and compact size, making it suitable for growth under laboratory conditions. The genome of rice, a model for monocots as well as an important cereal for staple food supply, was also one of the first to be analyzed (International Rice Genome Sequencing Project 2005). Since then, the genome sequences of several agronomically important crops, including vegetables, fruiting trees, and ornamental flowers, were analyzed, mostly for breeding purposes (Marks *et al.* 2021). Subsequently, the genomes of several non-crop wild plant species, including weeds, have been sequenced (Marks *et al.* 2021). Although this genome information has been used as “references” to understand the genetic and genomic bases of these species, these references were found to be insufficient to understand the genetics and genomics of these species. This issue has been partially addressed by resequencing analyses of the genomes of multiple individuals or cultivars of several species, including the Arabidopsis (The 1001 Genomes Consortium 2016) and rice (Wang *et al.* 2018). However, single nucleotide polymorphism was a major target since it was difficult to detect other types of sequence variations, such as insertions and deletions, by the short-read sequencing technology.

## Plant genome sequences determined by next-generation sequencing technology

Advances in plant genomics have been associated with advances in sequencing technology. The genomes of Arabidopsis and rice were initially sequenced using the Sanger method (International Rice Genome Sequencing Project 2005, The Arabidopsis Genome Initiative 2000). In the genome projects, a clone-by-clone strategy was employed to determine genome sequences at the chromosome level. Due to high costs and limitations in techniques, however, the resultant genome sequences possess gap regions, in which nucleotide sequences were not determined. Next-generation sequencing (NGS) methods (Heather and Chain 2016) have introduced a paradigm shift in both genomics and biology in the mid to late 2000s. NGS technologies are based on massive parallel reactions of sequencing-by-synthesis (Illumina, San Diego, CA, USA) or sequencing-by-ligation (ThermoFisher Scientific, Waltham, MA, USA), yielding whole-genome shotgun sequence reads at an Mb- or Gb-scale during one experiment and at low cost. NGS enabled the sequencing of various non-model plants, including vegetables, fruiting trees, and ornamental flowers (Marks *et al.* 2021). Although the resultant genome assemblies were highly fragmented because of the short reads from the NGS technologies, this information was sufficient to list gene sets in the genomes. These sequences were subsequently expanded to chromosome levels by the development of scaffolding technologies, including optical mapping (Bionano Genomics, San Diego, CA, USA) and Hi-C (Dudchenko *et al.* 2017). These technologies enabled the chromosome-level or chromosome-scale assembly of the genomes of more than 100 plant species by 2021, although the quality of these chromosome-level assemblies varied in genome coverage and gap contents (Shirasawa *et al.* 2021). It might be difficult for the NGS and Sanger methods to assemble centromeric and peri-centromeric regions comprised of repetitive sequences like transposable elements and clusters of repeated ribosomal DNA genes. However, in case of tomato, for which the first chromosome-level genome assembly is established with the short-read sequencing technologies (The Tomato Genome Consortium 2012), a ribosomal DNA arrays spanning a 15 Mb stretch at the end of a chromosome has been finally assembled with a long-read sequencing technology as mentioned below (Shirasawa and Ariizumi 2024).

## Haplotype-resolved genome assembly

Most practical reference genome sequences are haploid genome sequence. Pure lines of autogamous diploid plants have highly homozygous genomes. Thus, in assembling the genomes of these homozygotes, it is unnecessary to distinguish between pairs of homologous chromosomes containing the same base sequences. In allogamous and/or polyploid plants, however, the genome assembly of highly heterozygous individuals complicate assembly graphs, increasing the numbers of misassemblies and redundant contigs (Mochizuki *et al.* 2023). Additionally, typical haploid genome assembly can generate mosaic sequences that are mixtures of two haplotypes with heterozygous regions in a single chromosome (Delorean *et al.* 2023). The assembly of genomes that does not accurately represent the true genome may be misleading in downstream analysis. Advances in sequencing and bioinformatics methods may allow the generation of haplotype-resolved genome sequences, even in allogamous plants, polyploid species, and interspecific hybrids.

## Plant genome sequences at the T2T level

Haploids have been shown effective for improving the accuracy of genome assembly. Because the Human Genome Project used diploid cells of multiple individuals, the assembled human genomes contained euchromatic gaps caused by differences in segmental duplications (International Human Genome Sequencing Consortium 2004). The T2T Consortium used a complete hydatidiform mole (CHM) derived from a specific individual (Nurk *et al.* 2022). CHM is a tumor that develops from an abnormal fertilized egg in which the nucleus of the egg disappears and the haploid genome of the sperm doubles. The homozygous CHM genome was sequenced using various methods, such as HiFi long-read technology (PacBio), ultralong-read sequencing (Oxford Nanopore Technologies), PCR-free sequencing (Illumina), high-throughput chromosome conformation (Arima Genomics, Carlsbad, CA, USA), optical maps (Bionano Genomics), and single-cell DNA template strand sequencing (10X Genomics, Pleasanton, CA, USA). The first T2T genome assembly in plants involved the assembly of two of the ten chromosomes in maize (Liu *et al.* 2020).

Through a search with a term of “telomere-to-telomere” to NCBI PubMed database (https://pubmed.ncbi.nlm.nih.gov), T2T genome assemblies have been found to date in as many as 121 assemblies of 65 plant species (Table 1). T2T genome assembly in plants has shed light on the “dark matter” not detected in the genome sequences determined by classical sequencing and bioinformatics technologies, e.g., repetitive sequences associated with centromeric and pericentromeric regions (Sato *et al.* 2023) and clusters consisting of highly repeated sequences like ribosomal DNA genes (Shirasawa and Ariizumi 2024). These findings suggest that sophisticated bioinformatics technology is essential to accelerate T2T genome assembly in plant species.

## Sequencing technologies and bioinformatics tools for T2T genome assembly

### Telomere-to-Telomere genome assembly

Scaffolding (grouping, ordering, and orienting contigs) has been shown necessary to improve continuity and generate telomere-to-telomere assembly. Various types of long-distance linkage information, including optical maps and Hi-C/Omni-C contact maps, are used to construct chromosome-level scaffolds. These are used in genome assembly projects that aim to generate high-quality reference genome assemblies for diverse species, such as the Darwin Tree of Life Project (The Darwin Tree of Life Project Consortium 2022) (https://www.darwintreeoflife.org/) and the Vertebrate Genomes Project (Rhie *et al.* 2021) (https://vertebrategenomesproject.org).

Optical mapping involves the use of a light microscope-based technique to physically locate nicking enzyme recognition sites in the genome to produce DNA sequence fingerprints (Schwartz *et al.* 1993, Yuan *et al.* 2020). Optical maps generated using optical mapping technologies, such as Bionano (Bionano Genomics), provide information on the physical location and relative separation of labeling sites and have been used to improve contiguity and validate the genome assembly (Yuan *et al.* 2020).

Hi-C/Omni-C reads and information on long-range interactions are also valuable for scaffolding (Burton *et al.* 2013) and for the phasing of haplotypes. This technology has an advantage over optical mapping, in that the latter requires a large amount of high-molecular-weight genomic DNA, whereas Hi-C/Omni-C requires only chromatin from a single individual and short-read sequencing. Hi-C/Omni-C scaffolding has therefore been adopted as a major strategy in many studies of T2T genome assembly. For example, Hi-C/Omni-C scaffolding has been performed using tools such as YaHS (Zhou *et al.* 2023) and SALSA (Ghurye *et al.* 2017, 2019).

Telomeres in assembled genome sequences are identified by detecting telomeric repeat sequences using tools such as a Telomere Identification toolKit (tidk) (Brown *et al.* 2023). A telomeric repeat (TTTAGGG)_n_, first detected in *Arabidopsis thaliana* (Richards and Ausubel 1988), has been widely identified in plant genomes (Fuchs *et al.* 1995). Telomeric repeats in many plant species have been collected in comprehensive databases such as TeloBase (Lyčka *et al.* 2024) (http://cfb.ceitec.muni.cz/telobase/). In addition to the telomere detection, identifying centromeric regions could also validate the completeness of the genome assemblies, for which CentroMiner implemented in quarTeT (Lin *et al.* 2023) and TRASH (Wlodzimierz *et al.* 2023) are available. These tools and databases are valuable for identifying telomeres and centromeres in assembled genome sequences.

### Haplotype-phased genome assembly

Most plant genomes are complex structures, being large in size, having a high repeat content, and having high heterozygosity and polyploidy. Haplotype phasing in plant genomes could be achieved with trio data, sequencing reads obtained from both parents of a target individual, and/or high-throughput chromosome conformation capture (Hi-C/Omni-C), the latter of which provide information on long-range chromatin interactions. Trio binning partitions the target long sequence reads from both parents, followed by two separate assemblies of the partitioned reads (Koren *et al.* 2018). On the other hand, because Hi-C interactions occur more frequently within the same chromosome rather than between different chromosomes, with interaction frequency being inversely related to genome distance (Lieberman-Aiden *et al.* 2009, Xu and Dixon 2020), the information on the long-range interactions from Hi-C/Omni-C is also used to phase haplotypes (Kronenberg *et al.* 2021). A combination of trio data and Hi-C/Omni-C, together with HiFi and ultra-long reads, could be useful for haplotype-phased assembly in Hifiasm (Cheng *et al.* 2021) and Verkko (Rautiainen *et al.* 2023).

### Repetitive sequence annotation

Continuous genome assemblies can be constructed and long repeats can be resolved using long-read sequencing and assembly technology. Repetitive sequences in assembled genomes can be detected by *de novo*, homology-based, and structure-based methods (Liao *et al.* 2023), for which the pipeline of RepeatModeler2 is available (Flynn *et al.* 2020). *De novo* detection methods identify repetitive sequences without relying on the structures of repeat elements or their similarity to known repetitive sequences, with one *de novo* detection strategy using high-frequency *k*-mers and space seed extension, RepeatScout (Price *et al.* 2005). Homology-based detection methods, RepeatMasker (https://www.repeatmasker.org), identify repetitive sequences based on their similarity with known repeats. Many repetitive sequences from a wide range of species have been stored in databases, such as Repbase (Bao *et al.* 2015, Jurka 1998) and Dfam (Storer *et al.* 2021). Most repeats, especially transposable elements, have specific structures, similar to those of proteins or non-coding domains (Liao *et al.* 2023), which could be annotated by LTRharvest (Ellinghaus *et al.* 2008).

### Structural and functional annotation of genes

The structures of protein-encoding genes can be predicted by four methods: 1) *ab initio*, 2) transcript-based, 3) homology-based prediction, and 4) integration method.

1) The *ab initio* methods, e.g., AUGUSTUS (Stanke *et al.* 2006) and GeneMark-ES (Lomsadze *et al.* 2005), predict gene structures based on information contained in the genomic sequence and statistical models, such as Hidden Markov Models. Extrinsic evidence can also be used to train the model.

2) Alignment of information on RNA-seq reads, expressed sequence tags, full-length cDNA sequences, and/or isoform transcriptime sequences, so-called Iso-Seq (PacBio), from the target species can be used as extrinsic evidence, improving the accuracy of gene prediction. Transcript-based methods of Trinity (Grabherr *et al.* 2011), StringTie (Pertea *et al.* 2015), and TransDecoder (https://github.com/TransDecoder/TransDecoder) predict gene structures based on transcripts assembled using short-read RNA-seq and full-length RNA from the target genome. This method is highly reliable because it uses mRNA information as evidence of expression. Since not all genes are expressed in a single tissue/organ, highly comprehensive gene annotation requires the collection of RNA-seq data from multiple tissues and developmental stages.

3) Homology-based prediction methods implemented in Spaln (Gotoh 2008, 2024) and GeMoMa (Keilwagen *et al.* 2016, 2018) are based on spliced alignments of protein sequences from closely related species with the target genome. A large number of protein sequences from a wide range of species have been published and deposited in public databases, such as RefSeq (O’Leary *et al.* 2016) and UniProt Knowledgebase (UniProt Consortium 2023). This method, however, is less sensitive when the only protein sequences available are from species that are evolutionarily distant from the target species.

4) Integration methods, for which EVidenceModeler (Haas *et al.* 2008) and GINGER (Taniguchi *et al.* 2023) are available, integrate gene structures from other predictive methods to construct consensus gene structures, with these structures being more accurate and comprehensive than those based on other methods.

Because each of these methods has advantages and disadvantages, combining different methods is essential for complete gene annotation. The gene prediction pipelines of BRAKER3 (Gabriel *et al.* 2024) and MAKER2 (Holt and Yandell 2011) could simplify the complicated procedure. Gene annotation using extrinsic data depends on the quality and quantity of these data. Gene prediction tools using deep learning have also been described, with gene annotation based on DNA sequences alone using deep learning showing high-quality gene prediction in eukaryotic genomes (Stiehler *et al.* 2020).

In addition to protein-encoding genes, non-coding RNAs (ncRNAs), including transfer RNAs, ribosomal RNAs, small nucleolar RNAs, micro RNAs, and small-interfering RNAs, have also been annotated with tools, such as tRNAscan-SE2.0 (Chan *et al.* 2021) and RNAmmer (Lagesen *et al.* 2007). RNA genes are usually annotated based on sequence- and structure-based alignments with known RNAs. ncRNAs should be annotated using Rfam, a database of ncRNAs (Kalvari *et al.* 2021), and Infernal, a tool for sequence- and structure-based RNA alignments (Nawrocki and Eddy 2013).

Functional annotation in general relies on similarities between sequences from the target genome and those with characterized function from other genomes. The accumulation of gene function data from diverse species has enabled the annotation of functional information on unknown genes with the function of thousands of known genes using local alignment search tools such as BLAST.

UniProtKB is a high-quality and comprehensive resource on protein sequences and functions of various species (UniProt Consortium 2023). To date, UniProtKB contains over 245 million entries (release 2024_03 of UniProtKB; https://www.uniprot.org/). Pfam is a database of protein families and domains that has been used to analyze protein sequences from novel genomes (Mistry *et al.* 2021). To date, Pfam 37.0 contains 21,979 entries (http://pfam.xfam.org/).

The Gene Ontology (GO) knowledge base provides a comprehensive summary of gene structure and function. Gene function in GO has been described using three types (aspects) of functional characteristics: molecular function (molecular-level activity of the gene product), cellular component (location of the gene product), and biological process (biological program accomplished by multiple molecular activities) (The Gene Ontology Consortium 2023).

The Kyoto Encyclopedia of Genes and Genomes (KEGG) is a resource that integrates various biological objects categorized into systems, genomic, chemical, and health information (Kanehisa *et al.* 2023). The KEGG provides 16 manually curated databases in these four categories: molecular interaction, reaction, and relation networks in the systems information category; genes and proteins in the genomic information category; chemical compounds and reactions in the chemical information category; and human diseases and drugs in the health information category (Kanehisa *et al.* 2023).

The continued accumulation of sequence data and evidence from molecular, biochemical, and biophysical experiments, coupled with advances in informatics analysis technologies, is expected to facilitate comprehensive and accurate annotation of sequences and genes.

## Validation of the accuracy of genome assembly

Following the completion of assembly using advanced sequencing and bioinformatics technologies, the accuracy of the sequences should be validated to confirm that these sequences represent the actual genome structure. The N50 value is an indicator of continuity in draft genomes but is influenced by chromosome length. Therefore, the N50 values become meaningless in the chromosome-level assemblies.

One frequent validation method is bioinformatics metrics of BUSCO to assess genome assembly and annotation completeness with single-copy orthologs (Simão *et al.* 2015), Merqury to evaluate assembly quality with *k*-mer spectrum of whole genome sequencing reads (Rhie *et al.* 2020), and LAI to indicate the assembly quality of the intergenic and repetitive sequence space by the amount of identifiable intact long-terminal repeat elements (Ou *et al.* 2018). QUAST is also a quality assessment tool for evaluating and comparing genome assemblies providing reports, summary tables and plots on contig sizes, missassemblies and structural variations, genome representation and its functional elements, and variations of the N50 based on aligned blocks (Gurevich *et al.* 2013).

Alternative validation method is genetic mapping. Specifically, a genetic map is constructed based on the frequency of recombination between markers using a mapping population, thereby showing the relative positions of markers on chromosomes. Based on comparison between the positions of markers on genetic maps and on the assembled contigs, contigs can be ordered correctly and oriented to reconstruct chromosome-level scaffolds (Fierst 2015, Tang *et al.* 2015).

One importance drawback of genetic mapping is the requirement for mapping populations, especially in plants. It is not always possible to generate mapping population in plants. Woody plants require a long time to generate progenitors, and self-incompatible species require multiple individuals. These drawbacks may be resolved by using gametophytes, i.e., pollens in plants. Because each pollen possesses a haploid genome, consisting of recombinant chromosomes from the parent, each pollen could be recognized as an individual in a mapping population. Mapping based on haploid genomes in pollen has a great advantage over mapping based on diploid and polyploid genomes. Genotyping of a haploid requires only 1× coverage because of the absence of heterozygosity, although genotyping by sequencing generally requires 30× coverage. A tool, Scaffold Extender with Low Depth Linkage Analysis (SELDLA), has been developed to make linkage maps from low coverage data. A hybrid with distinguishable parent genomes can be regarded as the equivalent of two haploid organisms. SELDLA has been used to construct a linkage map of hybrids of two fish species, *Takifugu rubripes* and *Takifugu stictonotus*, from an average of 1.8× coverage data (Yoshitake *et al.* 2018). However, it may not be possible to prepare a sufficient population of organisms that are difficult to breed or cross, and more individuals may be required to construct a high-density linkage map. To solve these problems, a linkage map was constructed with sperm from of *Gasterosteus nipponicus* (Yoshitake *et al.* 2022). A single-cell DNA library was constructed using Chromium (10x Genomics, Pleasanton, CA, USA) and a high-density linkage map was constructed from an average of 0.13× coverage data by SELDLA. Multiple displacement amplification, which efficiently amplifies a small amount of DNA, may also amplify the small amount of genomic DNA in a single pollen, such that it is sufficient for genotyping. The resultant high-density linkage maps would be useful for validation of assembled sequences by determining the positions and/or directions of contigs.

## Future perspectives

Pan-genome analysis with T2T genome assembly can enhance understanding of the genetic and genomic components of target species. To date, 4,731 genome sequences are available for 1,875 plant species (by a search with a term of “Magnoliopsida” to NCBI Genome database; https://www.ncbi.nlm.nih.gov/datasets/genome), and T2T assemblies have been completed for 121 genomes across 65 species (Table 1). However, while 111 of them have simple genomes, e.g., diploid and doubled haploid, only 10 plants possess complex genomes of allopolyploid and interspecific hybrid. No T2T assembles has been so far reported for autopolyploidy. Moreover, >99% species in the plant kingdom have not yet been sequenced (Vallée *et al.* 2016). Furthermore, as Marks *et al.* (2021) mentioned, there are substantial taxonomic gaps in the current plant genome research and are numerous disconnects between the native range of focal species and the national affiliation of the researchers studying them. Genomic information on plants that have not yet been employed for breeding and industrial purposes is required to understand and dissect the genetics and genomics of the species and utilize this information to enhance plant breeding. We propose that, by focusing on plants, animals, and microorganisms endemic to Japan, for instance, researchers in Japan shall describe the importance of the genetic diversity (Washoku BioGenome Consortium launched by Prof. S. Kuraku of National Institute of Genetics and his colleagues). This regional attempt should be expanded over the world under the concept of the Earth BioGenome Project to record all genetic and genomic information on the planet toward the future (Lewin *et al.* 2022). The challenge would open up neo pan-genomics that covers not only within a single species but also bridges plants, animals, and microorganisms. In addition to sequencing, data management, including curation, distribution, and maintenance, should also be considered, with artificial intelligence possibly being a new interface to handle massive amounts of genomic data in the era of pan-genomics.

## Author Contribution Statement

KS designed the structure of the manuscript. All authors wrote the manuscript and approved the final version.

## Figures and Tables

**Table 1.** Statistics of plant genomes sequenced at the telomere-to-telomere level

Plant species name	Accession name*^a^*	Basic chr. no.*^b^*	Zygosity of the accession	Ploidy level of the accession	Total length of assembled sequences*^c^*	Reference DOI
*Actinidia chinensis*	Donghong	29	Heterozygous	Diploid	608 Mb	10.1016/j.molp.2022.12.022
*Actinidia chinensis*	Hongyang	29	Heterozygous	Diploid	606 Mb and 600 Mb	10.1093/hr/uhac264
*Actinidia eriantha*	Midao 31	29	Heterozygous	Diploid	619 Mb and 612 Mb	10.1186/s43897-023-00052-5
*Actinidia latifolia*	Kuoye	29	Heterozygous	Diploid	641 Mb	10.1016/j.molp.2022.12.022
*Arabidopsis thaliana*	Col-0	5	Homozygous	Diploid	134 Mb	10.1016/j.gpb.2021.08.003
*Armoracia rusticana*	HD15	16	Heterozygous	Allotetraploid	610 Mb	10.1038/s41467-023-39800-y
*Brassica napus*	Xiang5A	19	Homozygous	Allotetraploid	1.0 Gb	10.1093/hr/uhad171
*Brassica rapa*	Chiifu-401-42	10	Homozygous	Diploid	425 Mb	10.1111/pbi.14015
*Capsicum annuum*	G1-36576	12	Homozygous	Doubled haploid	3.1 Gb	10.1038/s41467-024-48643-0
*Capsicum rhomboideum*	PI 645680	13	n.a.	Diploid	1.7 Gb	10.1038/s41467-024-48643-0
*Chaenomeles speciosa*	n.a.	17	Heterozygous	Diploid	632 Mb	10.1093/hr/uhad183
*Citrullus amarus*	PI 189225	11	Homozygous	Diploid	378 Mb	10.1038/s41588-024-01823-6
*Citrullus amarus*	PI 271769	11	Homozygous	Diploid	378 Mb	10.1038/s41588-024-01823-6
*Citrullus amarus*	PI 296341-FR	11	Homozygous	Diploid	381 Mb	10.1038/s41588-024-01823-6
*Citrullus amarus*	PI 482276	11	Homozygous	Diploid	382 Mb	10.1038/s41588-024-01823-6
*Citrullus amarus*	RCAT 055816	11	Homozygous	Diploid	380 Mb	10.1038/s41588-024-01823-6
*Citrullus colocynthis*	PI 525081	11	Homozygous	Diploid	379 Mb	10.1038/s41588-024-01823-6
*Citrullus colocynthis*	PI 537300	11	Homozygous	Diploid	380 Mb	10.1038/s41588-024-01823-6
*Citrullus colocynthis*	PI 632755	11	Homozygous	Diploid	379 Mb	10.1038/s41588-024-01823-6
*Citrullus colocynthis*	PI 652554	11	Homozygous	Diploid	361 Mb	10.1038/s41588-024-01823-6
*Citrullus ecirrhosus*	PI 673135	11	Homozygous	Diploid	402 Mb	10.1038/s41588-024-01823-6
*Citrullus lanatus*	AllSugar	11	Homozygous	Diploid	369 Mb	10.1038/s41588-024-01823-6
*Citrullus lanatus*	Calhoun Gray	11	Homozygous	Diploid	370 Mb	10.1038/s41588-024-01823-6
*Citrullus lanatus*	Charleston Gray	11	Homozygous	Diploid	369 Mb	10.1038/s41588-024-01823-6
*Citrullus lanatus*	DBHZGua	11	Homozygous	Diploid	370 Mb	10.1038/s41588-024-01823-6
*Citrullus lanatus*	G42	11	Homozygous	Diploid	369 Mb	10.1016/j.molp.2022.06.010
*Citrullus lanatus*	G42	11	Homozygous	Diploid	369 Mb	10.1038/s41588-024-01823-6
*Citrullus lanatus*	HeiShanRen	11	Homozygous	Diploid	371 Mb	10.1038/s41588-024-01823-6
*Citrullus lanatus*	PI 254622	11	Homozygous	Diploid	374 Mb	10.1038/s41588-024-01823-6
*Citrullus lanatus*	PI 288522	11	Homozygous	Diploid	368 Mb	10.1038/s41588-024-01823-6
*Citrullus lanatus*	PI 381740	11	Homozygous	Diploid	370 Mb	10.1038/s41588-024-01823-6
*Citrullus lanatus*	PKR6	11	Homozygous	Diploid	371 Mb	10.1038/s41588-024-01823-6
*Citrullus lanatus*	SanBaiGua	11	Homozygous	Diploid	370 Mb	10.1038/s41588-024-01823-6
*Citrullus lanatus*	ShiHong No. 2	11	Homozygous	Diploid	369 Mb	10.1038/s41588-024-01823-6
*Citrullus lanatus*	Sugarlee	11	Homozygous	Diploid	369 Mb	10.1038/s41588-024-01823-6
*Citrullus mucosospermus*	PI 532732	11	Homozygous	Diploid	371 Mb	10.1038/s41588-024-01823-6
*Citrullus mucosospermus*	PI 595203	11	Homozygous	Diploid	371 Mb	10.1038/s41588-024-01823-6
*Citrullus naudinianus*	PI 596694	11	Homozygous	Diploid	365 Mb	10.1038/s41588-024-01823-6
*Citrullus rehmii*	PI 670011	11	Homozygous	Diploid	414 Mb	10.1038/s41588-024-01823-6
*Cucumis melo*	Kuizilikjiz	12	Homozygous	Diploid	379 Mb	10.1093/hr/uhad189
*Cucumis melo*	PI511890	12	Homozygous	Diploid	375 Mb	10.1111/tpj.16705
*Daucus carota*	Kurodagosun	9	Homozygous	Diploid	430 Mb	10.1093/hr/uhad103
*Dianthus caryophyllus*	Baltico	15	Heterozygous	Diploid	564 Mb and 568 Mb	10.1093/hr/uhad244
*Echinochloa phyllopogon*	R511	18	Homozygous	Allotetraploid	1.0 Gb	10.1093/dnares/dsad023
*Eleocharis dulcis*	n.a.	111	Heterozygous	Diploid	493 Mb	10.1038/s41597-024-03717-y
*Ficus hispida*	n.a.	14	Heterozygous	Diploid	372 Mb	10.1093/hr/uhad257
*Fragaria vesca*	Hawaii 4	7	Homozygous	Diploid	221 Mb	10.1093/hr/uhad027
*Glycine max*	Lee	20	Homozygous	Diploid	1.0 Gb	10.1002/tpg2.20382
*Glycine max*	Williams 82	20	Homozygous	Diploid	1.0 Gb	10.1002/tpg2.20382
*Glycine max*	Wm82-NJAU	20	Homozygous	Diploid	1.0 Gb	10.1016/j.molp.2023.08.012
*Glycine max*	Yundou1	20	Homozygous	Diploid	1.0 Gb	10.1016/j.xplc.2024.100919
*Glycine soja*	Yesheng71	20	Homozygous	Diploid	1.0 Gb	10.1016/j.xplc.2024.100919
*Gossypium raimondii*	D5-3	13	Homozygous	Diploid	776 Mb	10.1038/s41588-024-01877-6
*Gynostemma pentaphyllum*	n.a.	11	n.a.	Diploid	599 Mb	10.1016/j.xplc.2024.100932
*Ipomoea cairica*	n.a.	15	Heterozygous	Diploid	733 Mb	10.1093/g3journal/jkac187
*Jasminum sambac*	n.a.	13	Heterozygous	Diploid	495 Mb	10.1093/jxb/erac464
*Lactuca sativa*	PKU06	9	Homozygous	Diploid	2.6 Gb	10.1016/j.xplc.2024.101011
*Lolium perenne*	Kyuss	7	Homozygous	Doubled haploid	2.3 Gb	10.1093/gbe/evab159
*Mangifera indica*	Irwin	20	Heterozygous	Diploid	365 Mb	10.1002/tpg2.20441
*Manihot esculenta*	Xinxuan 048	18	Heterozygous	Diploid	665 Mb	10.1093/hr/uhad200
*Momordica charantia*	Jin ling zi or Lai pu tao	11	Homozygous	Diploid	296 Mb	10.1093/hr/uhac228
*Morella rubra*	Zaojia	8	Heterozygous	Diploid	293 Mb	10.1093/hr/uhae033
*Morus notabilis*	n.a.	6	Heterozygous	Diploid	410 Mb	10.1093/hr/uhad111
*Musa acuminata*	Baxijiao	11	Heterozygous	Triploid	477 Mb, 477 Mb, and 470 Mb	10.1093/hr/uhad153
*Musa acuminata*	DH-Pahang	11	Homozygous	Doubled haploid	485 Mb	10.1038/s42003-021-02559-3
*Musa acuminata*	n.a.	11	Heterozygous	Diploid	470 Mb and 470 Mb	10.1038/s41597-023-02546-9
*Oldenlandia diffusa*	n.a.	16	Homozygous	Diploid	500 Mb	10.1093/dnares/dsae012
*Olea europaea*	Leccino	23	n.a.	Diploid	1.3 Gb	10.1093/hr/uhae168
*Oryza barthii*	NH278	12	Homozygous	Diploid	350 Mb	10.1111/jipb.13607
*Oryza barthii*	NH279	12	Homozygous	Diploid	349 Mb	10.1111/jipb.13607
*Oryza barthii*	NH280	12	Homozygous	Diploid	349 Mb	10.1111/jipb.13607
*Oryza barthii*	NH281	12	Homozygous	Diploid	351 Mb	10.1111/jipb.13607
*Oryza barthii*	NH283	12	Homozygous	Diploid	349 Mb	10.1111/jipb.13607
*Oryza barthii*	NH284	12	Homozygous	Diploid	348 Mb	10.1111/jipb.13607
*Oryza barthii*	NH285	12	Homozygous	Diploid	348 Mb	10.1111/jipb.13607
*Oryza glaberrima*	NH266	12	Homozygous	Diploid	344 Mb	10.1111/jipb.13607
*Oryza glaberrima*	NH267	12	Homozygous	Diploid	347 Mb	10.1111/jipb.13607
*Oryza glaberrima*	NH268	12	Homozygous	Diploid	346 Mb	10.1111/jipb.13607
*Oryza glaberrima*	NH269	12	Homozygous	Diploid	348 Mb	10.1111/jipb.13607
*Oryza glaberrima*	NH270	12	Homozygous	Diploid	347 Mb	10.1111/jipb.13607
*Oryza glaberrima*	NH271	12	Homozygous	Diploid	345 Mb	10.1111/jipb.13607
*Oryza glaberrima*	NH272	12	Homozygous	Diploid	346 Mb	10.1111/jipb.13607
*Oryza glaberrima*	NH273	12	Homozygous	Diploid	346 Mb	10.1111/jipb.13607
*Oryza glaberrima*	NH274	12	Homozygous	Diploid	344 Mb	10.1111/jipb.13607
*Oryza glaberrima*	NH275	12	Homozygous	Diploid	348 Mb	10.1111/jipb.13607
*Oryza sativa*	HN	12	Homozygous	Diploid	394 Mb	10.1111/pbi.13880
*Oryza sativa*	J4155S	12	Homozygous	Diploid	395 Mb	10.1111/pbi.13880
*Oryza sativa*	LK638S	12	Homozygous	Diploid	396 Mb	10.1111/pbi.13880
*Oryza sativa*	XL628S	12	Homozygous	Diploid	398 Mb	10.1111/pbi.13880
*Oryza* sp.	NH277	12	Homozygous	Diploid	347 Mb	10.1111/jipb.13607
*Oryza* sp.	NH282	12	Homozygous	Diploid	348 Mb	10.1111/jipb.13607
*Panax ginseng*	n.a.	24	Heterozygous	Allotetraploid	3.5 Gb	10.1093/hr/uhae107
*Panicum miliaceum*	AJ8	18	n.a.	Allotetraploid	835 Mb	10.1038/s41597-024-03489-5
*Penthorum chinense*	n.a.	9	Homozygous	Diploid	258 Mb	10.1093/hr/uhad274
*Persea americana*	West Indian	12	Heterozygous	Diploid	842 Mb	10.1093/hr/uhae119
*Peucedanum praeruptorum*	n.a.	11	Heterozygous	Diploid	1.8 Gb	10.1093/gigascience/giae025
*Phragmites australis*	CN	25	n.a.	Allotetraploid	920 Mb	10.1038/s42003-024-06660-1
*Populus alba × Populus tremula*	84K	19	Heterozygous	Interspecific hybrid	417 Mb and 400 Mb	10.1093/plphys/kiae078
*Populus tremula × Populus alba*	INRA 717-1B4	19	Heterozygous	Interspecific hybrid	394 Mb and 403 Mb	10.1111/tpj.16454
*Prunus salicina*	Fengtangli	8	Heterozygous	Diploid	251 Mb and 251 Mb	10.1093/hr/uhae109
*Pyrus pyrifolia*	Yunhong No. 1	17	Heterozygous	Diploid	501 Mb	10.1093/hr/uhad201
*Quercus variabilis*	n.a.	12	Heterozygous	Diploid	789 Mb and 768 Mb	10.3389/fpls.2023.1290913
*Scutellaria baicalensis*	n.a.	9	Heterozygous	Diploid	385 Mb	10.1093/hr/uhad235
*Sesbania cannabina*	LJ5	12	n.a.	Allotetraploid	2.1 Gb	10.1007/s11427-023-2463-y
*Sorghum bicolor*	BTx623	10	Homozygous	Diploid	720 Mb	10.1002/imt2.193
*Sorghum bicolor*	Cuohu Bazi	10	Homozygous	Diploid	725 Mb	10.1038/s41597-024-03664-8
*Sorghum bicolor*	Hongyingzi	10	Homozygous	Diploid	746 Mb	10.1016/j.xplc.2024.100933
*Sorghum bicolor*	Huandiaonuo	10	Homozygous	Diploid	739 Mb	10.1016/j.xplc.2024.100933
*Sorghum bicolor*	Ji2055	10	Homozygous	Diploid	723 Mb	10.1002/imt2.193
*Theobroma grandiflorum*	1074	10	Heterozygous	Diploid	423 Mb	10.1093/gigascience/giae027
*Vaccinium duclouxii*	SGLD20220023	12	Heterozygous	Diploid	574 Mb	10.1093/hr/uhad209
*Vigna unguiculata*	Fengchan 6	11	Homozygous	Diploid	521 Mb	10.1111/pbi.14142
*Vitis* sp.	Thompson Seedless	19	Heterozygous	Diploid	505 Mb	10.1093/hr/uhad260
*Vitis vinifera*	Chasselas	19	Heterozygous	Diploid	500 Mb	10.1073/pnas.2403750121
*Vitis vinifera*	PN40024	19	Homozygous	Diploid	495 Mb	10.1093/hr/uhad061
*Vitis vinifera*	Ugni Blanc	19	Heterozygous	Diploid	495 Mb	10.1073/pnas.2403750121
*Vitis vinifera*	Yan73	19	Heterozygous	Diploid	502 Mb and 493 Mb	10.1093/hr/uhad205
*Zea mays*	B73-Ab10	10	Homozygous	Diploid	2.2 Gb	10.1186/s13059-020-02029-9
*Zea mays*	Mo17	10	Homozygous	Diploid	2.2 Gb	10.1038/s41588-023-01419-6
*Ziziphus jujuba*	Junzao	12	Heterozygous	Diploid	386 Mb	10.1093/hr/uhae071
*Ziziphus jujuba*	SZ	12	Heterozygous	Diploid	375 Mb	10.1093/hr/uhae071

*^a^* n.a. indicates not available. *^b^* Basic chromosome number (n). *^c^* Multiple values indicate haplotype-resolved assembly.
